# Serum LDH Levels in Normotensive and Preeclamptic-Eclamptic Pregnant Women and Its Correlation With Fetomaternal Outcome

**DOI:** 10.7759/cureus.37220

**Published:** 2023-04-06

**Authors:** Manila Reddy Eleti, Manjusha Agrawal, Deepika Dewani, Nidhi Goyal

**Affiliations:** 1 Department of Obstetrics and Gynecology, Jawaharlal Nehru Medical College, Datta Meghe Institute of Higher Education and Research (Deemed to be University), Wardha, IND

**Keywords:** low birth weight, abruptio placenta, serum ldh, eclampsia, preeclampsia

## Abstract

Background

One of the most prevalent medical issues observed during pregnancy is hypertension. Hypertensive disorders of pregnancy (HDP) and their consequences affect around 5-10% of all pregnancies globally. Preeclampsia is caused by endothelial dysfunction, which causes widespread endothelial leakage and contributes to potentially fatal consequences, such as eclampsia, placental abruption, disseminated intravascular coagulation (DIC), severe renal failure, pulmonary edema, and hepatocellular necrosis. As a result, looking for predictive markers for at-risk pregnancies that can suggest poor maternal or fetal outcomes is critical. Elevated levels of lactate dehydrogenase (LDH), as a sign of cellular damage and dysfunction, can be utilized as a biochemical marker in pregnancy-induced hypertension (PIH) as it represents the severity of the disease, and the occurrence of problems, and has also been demonstrated to co-relate with fetomaternal outcomes.

Methodology

A total of 230 singleton pregnant women of 28-40 weeks of gestational age were enrolled in this study. All women were divided into two groups - normotensive and preeclamptic-eclamptic groups; the second group was further divided into mild preeclampsia, severe preeclampsia, and eclampsia, based on blood pressure and the presence of proteinuria. Serum lactate dehydrogenase levels were measured in both groups and correlated with their fetomaternal outcome.

Results

Mean serum lactate dehydrogenase (LDH) level in eclamptic women was 1515.86 ± 754, in severely preeclamptic women was 932.2 ± 448, mild preeclamptic women were 580.5±213, while in normotensive women mean LDH level was 378.6 ± 124. The difference between normotensive and preeclamptic-eclamptic women was statistically significant (p < 0.001). The complications in the preeclamptic-eclamptic group were increased significantly in women with LDH > 800 IU/L, 600-800 IU/L compared to those who had < 600 IU/L LDH levels.

Conclusions

Serum LDH levels were significantly higher in women of preeclamptic-eclamptic group compared to the normotensive pregnant women. Higher LDH levels were positively correlated with disease severity and maternal complications like placental abruption, hemolysis elevated liver enzymes low platelet count (HELLP), disseminated intravascular coagulation (DIC), acute renal failure, intracranial hemorrhage, pulmonary edema, and maternal death and for fetal complications like preterm, intrauterine growth restriction (IUGR), APGAR at 1 minute < 7, APGAR at 5 minutes < 7, low birth weight (LBW), neonatal intensive care unit (NICU) admission and intrauterine fetal death (IUFD).

## Introduction

One of the most prevalent medical issues observed during pregnancy is hypertension. Hypertensive disorders of pregnancy (HDP) and their consequences affect around 5-10% of all pregnancies globally [1]. The incidence is also rising, owing primarily to older age at first pregnancy and increased pre-pregnancy weight [2]. Pregnancy hypertension is a major public health concern and one of the leading causes of maternal and perinatal morbidity and mortality [3]. It is estimated that pregnancy-induced hypertension (PIH) and associated complications account for approximately 14.0% of maternal deaths worldwide, necessitating additional immediate interventions for early detection and effective management of the problem in order to reduce maternal and perinatal outcomes [4].

Preeclampsia is caused by endothelial dysfunction, which causes widespread endothelial leakage and contributes to potentially fatal consequences, such as eclampsia, placental abruption, disseminated intravascular coagulation (DIC), severe renal failure, pulmonary edema, and hepatocellular necrosis [5-8]. Therefore, it is essential to look for predictive markers for at-risk pregnancies that can indicate poor maternal or fetal outcomes.

Lactate generation and high glucose consumption are common in the human placenta, and glycolysis is an important energy pathway [9]. Hypoxia stimulates metabolic pathways, strengthening glycolysis and raising lactate dehydrogenase (LDH) activity, which converts pyruvate to lactate [10]. LDH is secreted as an intracellular enzyme that is highly sensitive and can be used to diagnose a variety of illnesses in which cellular integrity is compromised. Gene expression and lactate dehydrogenase activity are higher in the preeclampsia placenta than in normal pregnancy [11]. Hypoxia increases LDH isoenzyme activity in trophoblasts, resulting in increased lactate generation. LDH has five isoforms, with LDH type 4 being the most vulnerable to hypoxia and prevalent in the placenta. It is found in the placenta of preeclampsia patients.

Elevated levels of LDH, as a sign of cellular damage and dysfunction, can be utilized as a biochemical marker in PIH since it represents the severity of the disease, the prevalence of complications, and has also been demonstrated to correlate with fetomaternal outcomes. Certain consequences of PIH, such as abruptio placentae, hemolysis elevated liver enzymes low platelet count (HELLP) syndrome, and renal failure, when cellular disintegration occurs, have elevated LDH values [11-13].

## Materials and methods

The sample size for this study was estimated using the prevalence of preeclampsia of 7.8% as reported in the study by Sajith et al. in 2014 with a 95% confidence interval and a 5% margin of error at a power of 80% and two-tailed test using the following formula n = Z_α/2 _× P × (1-P)/d^2 ^[14]. Here, Z_α/2_ is the level of significance at 95%, i.e., 95% confidence interval = 1.96, P = prevalence of hypertensive (HT) disorder in pregnancy = 7.8% = 0.08 (after rounding), d = desired error of margin = 5% = 0.05, n = 1.962 × 0.08 × (1-0.08)/0.052 = 113 patients needed in each group, preeclampsia-eclampsia and normotensive group, so the total sample size will be 226 patients. The minimum estimated sample size for the present study was 226. The minimum estimated sample size for the present study was 226. A total of 230 antenatal women, divided into normotensive (115) and preeclamptic-eclamptic (115) pregnant women, were studied in the Department of Obstetrics and Gynecology, Acharya Vinoba Bhave Rural Hospital (AVBRH), Wardha, India, between December 2020 and November 2022. The total number of deliveries during the same period was 3783.

**Figure 1:**
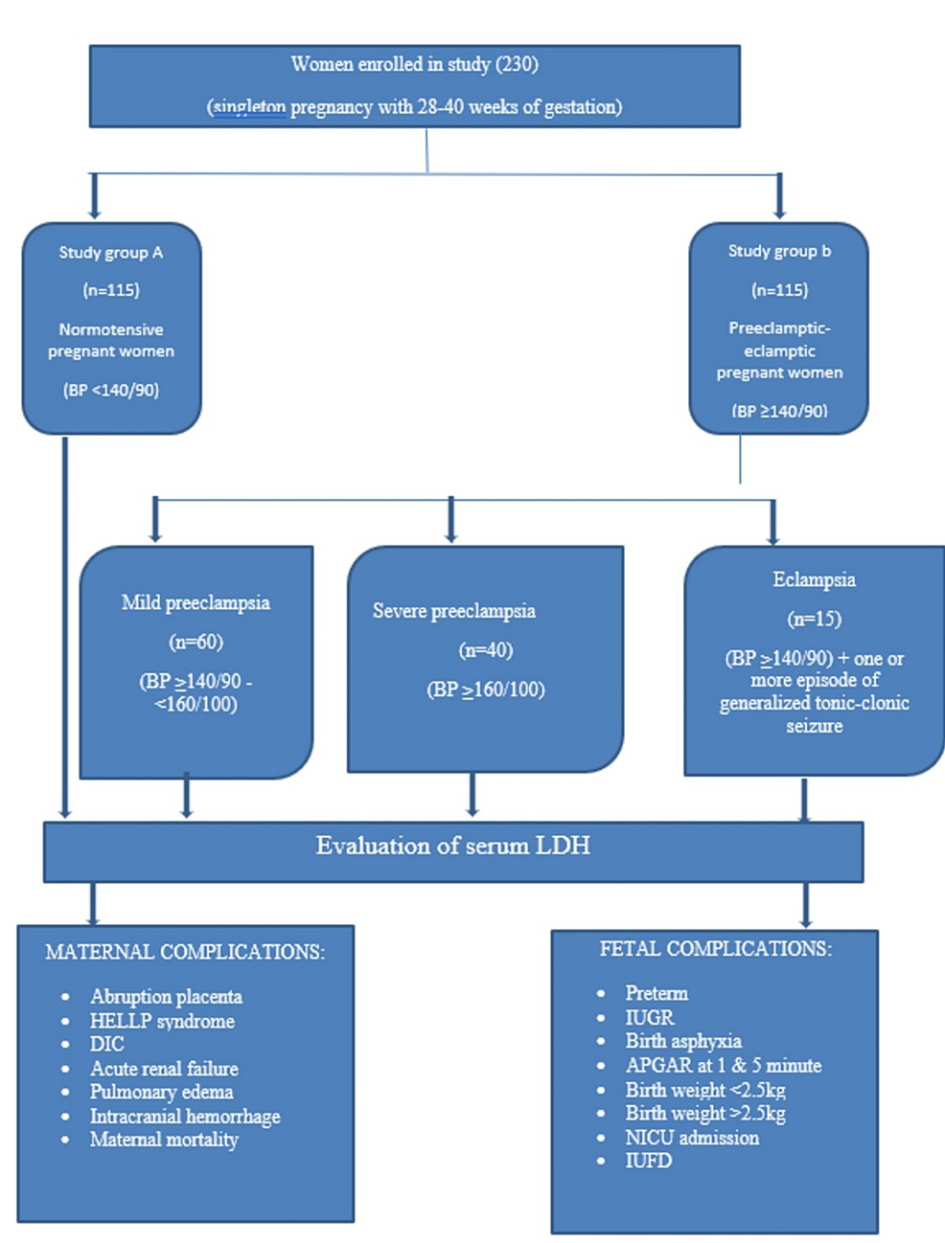
Schematic representation of the study design and methodology. BP: blood pressure; HELLP: hemolysis elevated liver enzymes low platelet count; DIC: disseminated intravascular coagulation; IUGR: intrauterine growth restriction; APGAR: activity, pulse, grimace, appearance, respiration; IUFD: intrauterine fetal death

Antenatal cases between 18 and 35 years, gestational age of 28-40 weeks, singleton pregnancy, normotensive, and preeclampsia-eclampsia women were included in this study. Mothers with hypertension at 20 weeks gestation (chronic hypertension), multiple pregnancies, pre-existing diabetes mellitus, liver disorder, renal disorder, epileptic disorder, thyroid disorder, heart illness, leukemia, hemolysis, hepatitis, and pancreatitis were excluded from this study. The study methodology is shown in Figure 1.

The women were divided into the following two groups: normotensive pregnant women (n = 115) and preeclamptic-eclamptic pregnant women (n = 115). Group II was classified into three categories as follows: mild preeclampsia (n = 60), severe preeclampsia (n = 40), and eclampsia (n = 15). Age, gravidity, parity, gestational age, and socioeconomic level were used to match the two groups. LDH levels of 600 IU/L are frequent in normal pregnancy, while levels of LDH > 600 IU/L have been linked to preeclamptic-eclamptic pregnant women. The women with preeclampsia and eclampsia were split into three groups based on their lactate dehydrogenase levels (600, 600-800, and >800 IU/L) to identify the group at high risk of developing maternal and fetal complications [13].

Technical procedure

Two milliliter of venous blood sample was collected from antecubital vein under all aseptic precautions in a plain bulb. Clotting of the sample was allowed for 30 minutes then it was centrifuged at 3000 rpm for 3 minutes for separating the serum for estimating the serum LDH and uric acid levels.

Serum LDH

**Figure 2:**
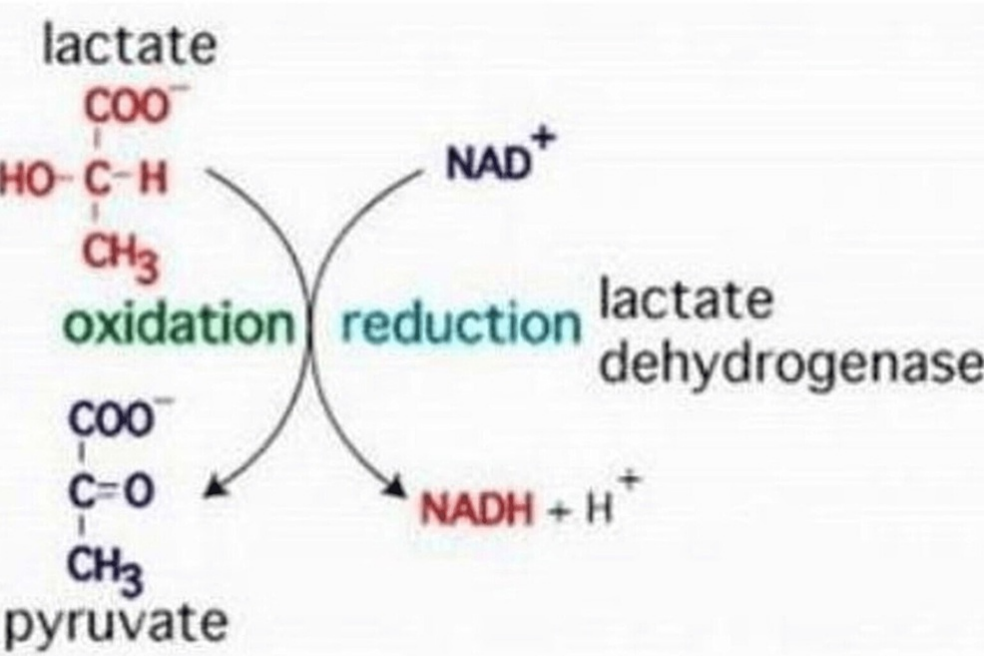
Conversion of pyruvate to lactate by LDH. LDH: lactate dehydrogenase

Estimation of serum LDH was done by enzymatic method on an automated clinical chemistry analyzer (VITROS 5600) using dry chemistry. Reduction of pyruvate with NADH forms NAD under lactate dehydrogenase enzyme that acts as a catalyst (Figure 2). It takes place at 30±0.050°C and a pH of 9.40±0.05. Maximum absorbance of NADH is seen at 340 nm, and after its exhaustion, the absorbance is reduced. This gives us the level of enzyme activity as detected by spectrophotometer.

## Results

As shown in Table 1, majority of the women in normotensive and preeclamptic-eclamptic belonged to the age group of 21-25 years which is 39.1% in each group. However, there was no notable variance in age distribution among the two groups (p = 0.98). As shown in Table 2, majority of the women were primigravida in both normotensive and preeclamptic-eclamptic. However, there was no unremarkable variance in parity distribution among the two groups (p = 0.98).

**Table 1:** Distribution of normotensive and preeclamptic-eclamptic women based on age (years) (n = 230).

Age group (years)	Normotensive (n = 115), no. (%)	Preeclamptic-eclamptic (n = 115)				χ^2 ^value; df; p-value
		Mild preeclampsia (n = 60) No. (%)	Severe preeclampsia (n = 40) No. (%)	Eclampsia (n = 15) No. (%)	Total (n = 115) No. (%)	χ^2^ = 0.1814 df = 3; p = 0.9805
≤20 years	9 (7.8)	2 (33.3)	3 (7.5)	3 (20.0)	8 (6.9)	
21-25 years	45 (39.1)	24 (40.0)	16 (40.0)	5 (33.3)	45 (39.1)	
26-30 years	44 (38.3)	23 (38.3)	14 (35.0)	6 (40.0)	43 (37.5)	
31-35 years	17 (14.8)	11 (18.3)	7 (17.5)	1 (6.7)	19 (16.5)	
Total	115 (100)	60 (100)	40 (100)	15 (100)	115 (100)	
Mean ± SD	26.10 ± 3.70	26.0 ± 4.20				

**Table 2:** Distribution of normotensive and preeclamptic-eclamptic women based on parity (n=230)

Parity	Normotensive	Preeclamptic-eclamptic (n = 115)				χ^2 ^value; df; p-value
	(n = 115), no. (%)	Mild preeclampsia (n = 60), no. (%)	Severe preeclampsia (n = 40), no. (%)	Eclampsia (n = 15), no. (%)	Total (n = 115), no. (%)	
Primigravida	53 (46.08)	21 (35.0)	24 (60.0)	9 (60.0)	54 (46.95)	χ^2^ = 0.1522; df = 3; p = 0.9849
G2	46 (40)	29 (48.3)	11 (27.5)	6 (40.0)	46 (40)	
G3	12 (10.43)	10 (16.7)	2 (5.0)	0 (0)	12 (10.43)	
≥G4	4 (3.47)	0 (0)	3 (7.5)	0 (0)	3 (2.60)	
Total	115 (100)	60 (100)	40 (100)	15 (100)	115 (100)	

Overall, as shown in Table 3, majority of the women belonged to the gestational age between 37 and 40 weeks. About 69% women in normotensive group and 64.3% in preeclamptic-eclamptic group had gestational age between 37 and 40 weeks. There was no significant difference between the two groups (p = 0.66).

**Table 3:** Distribution of normotensive and preeclamptic-eclamptic women based on gestational age (weeks) (n = 230).

Gestational age (weeks)	Normotensive	Preeclamptic-eclamptic (n = 115)				χ^2 ^value; df; p-value
	(n = 115), no. (%)	Mild preeclampsia (n = 60), no. (%)	Severe preeclampsia (n = 40), no. (%)	Eclampsia (n = 15), no. (%)	Total (n = 115), no. (%)	
28-32 + 6	5 (4.34)	1 (1.6)	2 (5.0)	4 (26.7)	7 (6.08)	χ2 = 0.817; df = 2; p = 0.66
33-36 + 6	30 (26.08)	13 (21.7)	16 (40.0)	5 (33.3)	34 (29.56)	
37-40	80 (69.56)	46 (76.7)	22 (55.0)	6 (40.0)	74 (64.34)	
Total	115 (100)	60 (100)	40 (100)	15 (100)	115 (100)	
Range	31-40	-	28.6-39.6		-	

As shown in Table 4, mean and standard deviation of systolic blood pressure (mmHg) in eclampsia was 170.93 ± 18.82, severe preeclampsia was 170.90 ± 9.75 and mild preeclampsia was 151.93 ± 5.04 which was significantly higher as compared to normotensive 122.33 ± 8.14. Mean and standard deviation of diastolic blood pressure (mmHg) in eclampsia was 111.20 ± 7, severe preeclampsia was 113.30 ± 3.34, and mild preeclampsia was 101.80 ± 4.33, which was significantly higher as compared to normotensive 79.1 ± 5.81.

**Table 4:** Distribution of normotensive and preeclamptic-eclamptic women based on systolic and diastolic blood pressure. *P-value is significant. SBP: systolic blood pressure; DBP: diastolic blood pressure

BP (mean and standard deviation)	Normotensive (n = 115)	Preeclamptic eclamptic (n = 115)			F-value/p-value
		Mild preeclampsia (n = 60)	Severe preeclampsia (n = 40)	Eclampsia (n = 15)	
SBP	122.33 ± 8.14	151.93 ± 5.04	170.90 ± 9.75	170.93 ± 18.82	F = 202.40/p = 0.0001*
DBP	79.1 ± 5.81	101.80 ± 4.33	113.30 ± 3.34	111.20 ± 7	F = 403.73/p = 0.0001*

As shown in Table 5, mean serum LDH in eclamptic women was 1515.86 ± 754, severe preeclamptic women was 932.2 ± 448, mild preeclamptic women was 580.5 ± 213, while in normotensive women mean LDH level was 378.6 ± 124. The difference between normotensive and preeclamptic-eclamptic women was statistically significant (p<0.001).

**Table 5:** Distribution of normotensive and preeclamptic-eclamptic women based on serum LDH levels (IU/L) (n = 230). LDH: lactate dehydrogenase

Serum LDH level (IU/L)	Normotensive (n = 115), no. (%)	Preeclamptic-eclamptic (n = 115)				Total (n = 230), no. (%)	χ^2 ^value; df; p-value
		Mild preeclampsia (n = 60), no. (%)	Severe preeclampsia (n = 40), no. (%)	Eclampsia (n = 15), no. (%)	Total (n = 115), no. (%)		
<600	113 (98.26)	41 (68.33)	9 (22.5)	1 (6.66)	51 (44.34)	164 (71.30)	χ^2^ = 126.6; df = 6; p < 0.0001*
600-800	2 (1.73)	13 (21.66)	15 (37.5)	5 (33.33)	33 (28.69)	35 (15.21)	
>800	0 (0)	6 (10)	16 (40)	9 (60)	31 (26.95)	31 (13.47)	
Total	115 (100)	60 (100)	40 (100)	15 (100)	115 (100)	230 (100)	
Mean ± SD	378.6 ± 124.9	580.56 ± 213.21	932.2 ± 448.28	1515.86 ± 754.1	-		
Range	112-675	375-1890	374-2430	578-2450			

As shown in Table 6, in normotensive group, most common mode of delivery was full-term vaginal delivery (58.2%), while in preeclamptic eclamptic group was lower segment cesarean section (40.8%). In preeclamptic-eclamptic group, lower segment cesarean section (LSCS) was observed significantly more in eclampsia group 60% of women as compared to severe preeclampsia (52%) and mild preeclampsia (28%). In eclamptic and severe preeclamptic groups, majority of women undergoing LSCS had serum LDH levels >800. The observed results were statistically significant (p < 0.001).

**Table 6:** Distribution of normotensive and preeclamptic-eclamptic women based on serum LDH levels and mode of delivery (n = 230). FTVD: full-term vaginal delivery; PTVD: preterm vaginal delivery; ID: instrumental delivery; LSCS: lower segment cesarean section; LDH: lactate dehydrogenase

Mode of delivery	Normotensive (n = 115), no. (%)				Preeclamptic-eclamptic (n = 115)										χ^2 ^value; df; p-value
					Mild preeclampsia (n = 60), no. (%)			Severe preeclampsia (n = 40), no. (%)			Eclampsia (n = 15), no. (%)			Total no. (%)	
	<600	600-800	>800	Total	<600	600-800	>800	<600	600-800	>800	<600	600-800	>800		
FTVD	67 (58.26)	0	0	67 (58.26)	24 (40)	3 (5)	3 (5)	4 (10)	4 (10)	5 (12.5)	0	1 (6.66)	0	44 (38.2)	χ^2^ = 49.65; df = 9; p < 0.001
PTVD	26 (22.6)	0	0	26 (22.6)	4 (6.6)	1 (1.66)	0	1 (2.5)	1 (2.5)	0	0	1 (6.66)	3 (20)	11 (9.56)	
ID	8 (6.95)	0	0	8 (6.95)	7 (11.6)	1 (1.66)	0	0	2 (5)	2 (5)	0	1 (6.66)	0	13 (11.3)	
LSCS	12 (10.43)	2 (1.78)	0	14 (12.17)	5 (8.33)	9 (15)	3 (5)	4 (10)	8 (20)	9 (22.5)	1 (6.66)	2 (13.33)	6 (40)	47 (40.8)	

Overall, as shown in Table 7, most common maternal complication observed was abruptio placenta, which was reported in 0.8% of women in normotensive group, 16% in preeclampsia group, and 33.3% in eclampsia group. A total of 5.8% of women had abruptio placenta at serum LDH < 600 IU/L, 24.2% at serum LDH 600-800 IU/L, and 32.2% at serum LDH > 800 IU/L in preeclamptic-eclamptic group.

**Table 7:** Distribution of normotensive and preeclamptic-eclamptic women according to the serum LDH levels and correlation with its maternal outcomes. HELLP: hemolysis elevated liver enzymes low platelet count; DIC: disseminated intravascular coagulation; LDH: lactate dehydrogenase

Maternal outcomes	Normotensive group (serum LDH levels)				Preeclamptic-eclamptic group (serum LDH levels)									
					Mild preeclampsia			Severe preeclampsia			Eclampsia			Total
	<600	600-800	>800	Total	<600	600-800	>800	<600	600-800	>800	<600	600-800	>800	
Abruptio placentae	1	0	0	1	1	0	0	2	6	7	0	2	3	21
HELLP syndrome	0	0	0	0	0	1	1	0	1	2	0	0	3	8
DIC	0	0	0	0	0	0	0	0	0	2	0	2	1	5
Acute renal failure	0	0	0	0	0	0	0	0	2	1	0	1	1	5
Pulmonary edema	0	0	0	0	0	0	0	0	0	1	0	0	0	1
Intracranial hemorrhage	0	0	0	0	0	0	0	0	0	1	0	0	0	1
Maternal death	0	0	0	0	0	0	0	0	0	1	0	0	0	1

As shown in Table 8, low birth weight was observed in 15.6% of women in normotensive group, whereas in preeclamptic-eclamptic it was seen in 49.5% of women. A total of 31.3% of women observed low birth weight with serum LDH < 600 IU/L, 33.3% at LDH 600-800 IU/L, and 96.7% at serum LDH > 800 IU/L in preeclamptic-eclamptic group.

**Table 8:** Distribution of normotensive and preeclamptic-eclamptic women according to the serum LDH levels and correlation with its fetal outcomes. IUGR: intrauterine growth restriction; APGAR: appearance, pulse, grimace, activity, respiration; IUFD: intrauterine fetal death; LDH: lactate dehydrogenase

Fetal outcomes	Normotensive group (serum LDH levels)				Preeclamptic-eclamptic group (serum LDH levels)									
					Mild preeclampsia			Severe preeclampsia			Eclampsia			Total
	<600	600-800	>800	Total	<600	600-800	>800	<600	600-800	>800	<600	600-800	>800	
Preterm	35	0	0	35	7	4	3	5	7	4	1	1	5	37
IUGR	11	1	0	12	7	8	2	6	9	9	0	2	4	47
Birth asphyxia	3	0	0	3	2	1	0	0	1	1	0	1	0	6
APGAR at 1 minute < 7	19	1	0	19	29	3	2	9	15	20	1	5	9	93
APGAR at 5 minutes < 7	9	1	0	9	1	2	0	5	1	7	0	0	4	20
Birth weight < 2.5kg	18	0	0	18	7	0	1	8	8	20	1	3	9	57
Birth weight > 2.5kg	95	2	0	95	28	6	0	1	7	1	0	2	0	45
NICU admission	9	1	0	10	5	7	2	8	12	11	0	1	5	51
IUFD	0	0	0	0	0	0	0	0	1	3	0	3	4	11

## Discussion

Age

In the present study, most of the participants, 90 (39.13%) patients, were in the age group of 21-25 years followed by 87 (37.8%) patients in the age group of 26-30 years. The mean age for normotensive pregnant women was 26 ± 10 years and for preeclamptic-eclamptic pregnant women was 26 ± 4.20 years. All the groups were similar with regard to their age distribution (p = 0.9805). A similar age range was reported by other studies [2,11,15].

Gupta et al. in 2019 reported that the maximum number of women belonged to the age group of 20-25 years followed by 25-30 years, the mean gestational age for the study group was 24.38 ± 3.68 years and the control group was 25 ± 2.99 years [16]. Similarly, Jaiswar et al. in 2011 reported that a maximum number of women belonged to the age group of 21-30 years, the mean age of control was 25.46 ± 3.29 years, and mild preeclampsia group was 25.80 ± 3.30 years, severe preeclampsia 26.03 ± 3.99 years, and eclampsia was 24.50 ± 3.45 years [17].

Parity

In the present out of a total of 230 patients, 107 (46.2%) were primigravida, 92 (40%) were second gravida, 24 (10.43%) were third gravida, and seven (3.04%) patients were fourth gravida and above in the normotensive and in the preeclamptic-eclamptic group. All the groups were similar with regards to their parity distribution which was not significant (p = 0.9849), although primigravida was higher as compared to second and multigravida.

Qublan et al. in 2005 observed that majority of the cases were young primigravidas who were affected by preeclampsia (PE) in the study population. Primigravida was noted in 16.6% of normotensive women, 26.5% of women with mild preeclampsia, and 53.2% of women with severe preeclampsia, similar to this study [18]. Similarly, Dave et al. in 2016 stated that the majority of the cases were primigravida, 61% in preeclampsia and 43% in the normotensive group followed by second gravida 28% in preeclampsia and 34% in the normotensive group [19]. Some of the studies conducted in other geographical locations also confirmed the higher incidence of PE among primigravida, such as those conducted in Egypt, Nigeria, and Uganda which declares primigravida as a risk factor for preeclampsia/eclampsia [20-22].

Gestational age

In this study out of a total of 230 patients, a significantly higher number of women, i.e., 80 (69.56%) in normotensive, 46 (76.7%) in mild preeclampsia, 22 (55%) in severe PE, and six (40%) in eclampsia group were from gestational age 37-40 weeks than 33-36+6 and 28-32+6 weeks. All the groups were similar with regard to their gestational age distribution which was not significant (p = 0.66). Prajapati and Maitra in 2013 reported that majority of pregnancy-induced hypertension cases, i.e., 46.6% were at gestational age of 37-39 weeks at the time of delivery, comparable with this study [2]. Similarly, Kumar et al. in 2019 reported that the majority of the women (51.43% and 48.98%) who were diagnosed with gestational hypertension and preeclampsia presented between 37 and 40 weeks, similar to this study [23].

Blood pressure

In the present study, it was observed that the mean systolic and diastolic blood pressure (BP) reading for eclampsia group was 170.93 ± 18.82 and 111.20 ± 7, for severe preeclampsia group it was 170.90 ± 9.75 and 113.30 ± 3.34, mild preeclampsia was 151.93 ± 5.04 and 101.80 ± 4.33, and for the normotensive group it was 122.33 ± 8.14 and 79.1 ± 5.81. Based on the findings of the study conducted by Bellomo et al. in 2011, among women diagnosed with preeclampsia, the mean systolic reading was 149.0 ± 11.7 mmHg while the diastolic reading was 96.1 ± 6.2 mmHg, which was not comparable with the present study [24,25]. Another study conducted by Sachan et al. in 2013 states that the mean SBP and DBP among severe preeclampsia cases were 159.88 ± 8.3 and 103.25 ± 7.6, respectively; for mild preeclampsia group, the mean SBP and DBP were 145.32 ± 9.7 and 93.72 ± 5.2, respectively; while for the controls, it was 117.84 ± 4.7 and 77.42 ± 6.8. The study depicted a significant difference in the mean SBP and DBP among the three groups [26].

LDH levels in normotensive and preeclamptic-eclamptic pregnant women

In the present study, it was observed that the LDH levels > 800 IU/L were significantly higher in eclampsia group in 9 (60%) patients, severe preeclampsia in 16 (40%) patients, mild preeclampsia group in six (10%) patients, and compared to none in the normotensive group (p < 0.0001). Similarly, LDH values between 600 and 800 IU/L were significantly higher in severe preeclampsia group in 15 (37.5%) patients, in eclampsia group in five (33.33%) patients, and in mild preeclampsia group in 13 (21.66%) patients as compared to normotensive group in two (1.73%) patients (p < 0.0001). Majority of the cases from the normotensive group had LDH levels < 600 IU, in 113 (98.26%) cases. The mean serum LDH levels in eclampsia was 1515.86 ± 754.13, in severe preeclampsia was 932.20 ± 448.28, in mild preeclampsia was 580.56 ± 213.21, and in normotensive was 389.05 ± 143.38.

The study by Mary et al. also observed a significant rise in LDH levels with increasing disease severity (p < 0.001). Mean LDH levels were 323 ± 58 in the control group, as compared to 478 ± 86 in those with mild preeclampsia and 756 ± 76 in cases with severe preeclampsia. The data obtained from the above study were not comparable with this study [13]. In another study by Jaiswar et al., mean LDH level in the control group was 278.3 ± 119.2 IU/L as compared to 400.45 + 145 IU/L in mild preeclamptic and 646.95 ± 401.64 IU/L in severe preeclamptic groups, and still higher levels with 1648.10 ± 1992.29 IU/L in the eclamptic group. Thus, the serum LDH levels showed a consistent and statistically significant increase with rising blood pressure levels both systolic and diastolic (p < 0.001) were comparable with this study [17]. In another study by Prajapati and Maitra in 2013 there was a notable rise in mean LDH levels with rising severity of disease from gestational hypertension 536 ± 178.75, mild preeclampsia 626.59 ± 225.72, severe preeclampsia 699.54 ± 254.17, to eclampsia 1270.63 ± 753.58 (p < 0.001 [2].

Mode of delivery

In the present study, full-term vaginal deliveries were more in normotensive group, 67 (58.26%) cases, than in preeclamptic-eclamptic group, 47 (40.86%) cases. The LSCS were more in preeclamptic-eclamptic group, 43 (37.39%) cases, compared to normotensive group, 14 (12.17%) cases. Among the preeclamptic-eclamptic group, the LSCS were more in eclampsia, nine (60%) cases, than in severe preeclampsia, 17 (42.5%) cases, and mild preeclampsia, 17 (28.33%) cases. LSCS rate was statistically significant in the preeclamptic-eclamptic group as compared to the normotensive group (p < 0.0001). Based on the findings of the study conducted by Bellomo et al. in 2011, among women diagnosed with preeclampsia, 48% of the women delivered via cesarean section [25]. Another study by Sachan et al. in 2013 stated similar findings where LSCS was performed for 43.75% of women with severe preeclampsia in comparison to the controls (32.26%) [26]. Another study by Qublan et al. stated that increasing levels of LDH has more rate of cesarean section (69.2%) and normal vaginal delivery (30.8%) [18].

Serum LDH and maternal complications

Among the maternal outcomes, one mother had intracranial hemorrhage (LDH > 800 IU/L), one had pulmonary edema (LDH > 800 IU/L) and one mother died postpartum due to abruptio placenta and HELLP syndrome (LDH > 800 IU/L). Out of 22 women, 10 had abruption with LDH more than 800 IU/L (p < 0.001), and out of eight women, six had HELLP (p = 0.031) with LDH levels more than 800 IU/L. Out of five cases of DIC, two were in the severe PE group and one in eclampsia (E) group with serum LDH levels of > 800 IU/L, and out of five cases of acute renal failure one each in severe PE and E group with serum LDH levels > 800 IU/L. Out of 22 women, eight had abruption with LDH 600-800 IU/L, six in severe PE, two in E group, and out of eight women two had HELLP syndrome with LDH 600-800 IU/L, one each in mild PE and severe PE group. Out of five women, three had DIC in the E group with LDH 600-800IU/L. Out of five women, three had acute renal failure with LDH 600-800IU/L two in severe PE, and one in the E group. Out of 22 women, two had abruption with LDH > 600 IU/L, one each in mild PE and normotensive group.

The findings of this study are similar to those from the study done by Jaiswar et al. which showed that only one case had abruptio placenta and one case had cerebrovascular accident with LDH levels of 600-800 IU/L, while eight instances had abruptio placenta, HELLP syndrome with renal failure (RF), metabolic encephalopathy, pulmonary embolism, pulmonary edema, and renal failure, and two cases had cerebrovascular accident with LDH levels > 800 IU/L. Similar to the current investigation, there was a statistically significant increase in maternal problems with increasing LDH levels (p < 0.001) [17].

Mary et al. also reported a higher incidence of maternal complications with rising LDH levels. In their study, among patients with LDH > 800 IU/L, 94.3% developed complications like eclampsia: seven (38.8%), abruption: four (22.2%), HELLP syndrome: two (11.1%), intracranial hemorrhage: one (5.5%), pulmonary edema: one (5.5%), acute renal failure: one (5.5%), and DIC: one (5.5%), as compared to 13.6% with LDH levels of 600-800 IU/L developed complications like eclampsia: two (6.8%), and abruption: one (3.4%), which was found to be statistically significant. They also observed that high LDH levels were associated with a statistically significant impairment of renal and liver function [13]. The data obtained from the above study were comparable with the present study, with LDH levels > 800 IU/L.

Prajapati and Maitra also reported a higher incidence of maternal complications with rising LDH levels [2]. Prajapati and Maitra reported a higher incidence of maternal complications among patients with LDH > 800 IU/L, such as eclampsia: 12 (36.3%), abruption: four (12.12%), HELLP syndrome: 20 (62.5%), acute renal failure: three (9.09%), DIC: two (6.06%), postpartum hemorrhage: one (3.03%), maternal IUC admission: eight (24.24%), maternal death: one (3.03%) with rising LDH levels; more than 2/3rd (62.5%) of cases with LDH level > 800 IU/L had complications (p < 0.001) [2]. In the same study with LDH levels 600-800 IU/L maternal complications like HELLP syndrome: four (8.33%), eclampsia: two (4.1%), abruption: two (4.1%), acute renal failure: four (8.33%), DIC: one (2.08%), pulmonary edema: two (4.1%), maternal IUC admission: two (4.16%) were seen; and with LDH levels < 600 IU/L complications like HELLP syndrome: two (2.02%), eclampsia: five, (5.05%), abruptio placenta: two (2.02%) were seen. As shown in Table 9, the present study LDH levels in preeclampsia group were comparable with studies of Dave et al. with LDH levels < 600IU/L, Prajapati and Maitra with LDH levels of 600-800 IU/L, and Gupta et al. with LDH levels > 800 IU/L [2,16,19].

**Table 9:** Correlation of serum LDH with maternal complications in preeclampsia with other studies. AP: abruptio placenta; HELLP: hemolysis elevated liver enzymes low platelet count; DIC: disseminated intravascular coagulation; ARF: acute renal failure

Authors	LDH < 600				LDH 600-800				LDH > 800			
	AP	HELLP	DIC	ARF	AP	HELLP	DIC	ARF	AP	HELLP	DIC	ARF
This study	3 (6%)	-	-	-	6 (21.4%)	2 (7.14%)	-	2 (7.14%)	7 (31.8%)	3 (13.6%)	2 (9.09%)	1 (4.54%)
Prajapati and Maitra in 2013 [2]	2 (2%)	2 (2%)	-	-	2 (4.1%)	4 (8.33%)	2 (4.1%)	4 (8.33%)	4 (12.12%)	16 (48.4%)	2 (6%)	3 (9%)
Gupta et al. in 2019 [16]	5 (9.4%)	-	-	-	3 (11.1%)	1 (3.7%)	1 (3.7%)	1 (3.7%)	6 (30%)	4 (20%)	2 (10%)	2 (10%)
Mary ​​​​​​et al. in 2017 [13]	-	-	-	-	1 (3.4%)	-	1 (3.4%)	-	4 (22.2%)	2(11.1%)	1 (5.5%)	1 (5.5%)
Dave et al. in 2016 [19]	1 (2.1%)	-	0	0	2 (5.5%)	-	11 (30.5%)	0	8 (14%)	-	36 (63%)	5 (8.7%)
Jaiswar et al. 2011 [17]	-	-	-	-	1 (7.7%)	-	-	-	1 (7.7%)	1 (7.7%)	-	1 (7.7%)

Serum LDH and fetal complications

In the normotensive group, there were 35 preterm births, 11 IUGR, three cases of birth asphyxia, 19 APGAR at 1 minute < 7, nine APGAR at 5 minutes < 7, 18 birth weight < 2.5 kg, 95 birth weight > 2.5 kg, and nine NICU admissions with serum LDH levels of < 600 IU/L, one IUGR, one NICU admission, and two birth weight > 2.5 kg with serum LDH levels of 600-800 IU/L. In the preeclamptic-eclamptic group, there were 37 preterm births, 47 IUGR, six birth asphyxia, 93 APGAR at 1 minute < 7, 20 APGAR at 5 minutes < 7, 57 birth weight < 2.5 kg, 45 birth weight > 2.5 kg, and 51 NICU admissions and 11 IUFD.

As shown in Table 10, Gupta et al. in 2019 observed that mean birth weight (kg) with serum LDH levels < 600 IU/L was 2.36 ± 0.60, with serum LDH levels 600-800 IU/L was 2.20 ± 0.52, and with serum LDH levels > 800 IU/L was 1.99 ± 0.59, most of the babies with serum LDH levels > 800 IU/L required NICU admission (50%) [16]. In this study in preeclampsia group, prematurity and LBW were noted in six (30%) and 17 (85%) cases, respectively, which were comparable with this study with prematurity noted in seven (31.8%) and LBW in 21 (95.45%) cases. In the study by Gupta et al. prematurity was noted in six (30%) and LBW in 17 (85%) cases in the preeclampsia group with LDH levels > 800 IU/L, this result was comparable with the present study with prematurity noted in seven (31.8%) and LBW in 21 (95.45%) cases [16]. In the study by Singh et al. in 2018 prematurity was noted in 13 (34.5%) and low birth weight in nine (28.1%) cases in the preeclampsia group with LDH levels of 600-800 IU/L, this result was comparable with the present study with prematurity noted in 11 (39.2%) and low birth weight in eight (28.5%) cases [27].

**Table 10:** Correlation of serum LDH with fetal complications in preeclampsia with other studies. IUGR: intrauterine growth restriction; LBW: low birth weight; IUFD: intrauterine fetal death; LDH: lactate dehydrogenase

Authors	LDH < 600				LDH 600-800				LDH > 800			
	Preterm	IUGR	LBW	IUFD	Preterm	IUGR	LBW	IUFD	Preterm	IUGR	LBW	IUFD
This study	12 (24%)	13 (26%)	15 (30%)	0(0%)	11 (39.2%)	17 (60.7%)	8 (28.5%)	1 (3.57%)	7 (31.8%)	11 (50%)	21 (95.45%)	3 (13.6%)
Lavanya et al. in 2022 [15]	1 (2%)	4 (7.8%)	1 (2%)	1 (2%)	0 (0%)	1 (3.4%)	1 (3.4%)	0 (0%)	0 (0%)	3 (15%)	0 (0%)	0 (0%)
Prajapati and Maitra in 2013 [2]	-	18 (18.18%)	-	4 (4.04%)	-	15 (31.25%)	-	3 (6.25%)	-	14 (42.42%)	-	7 (21.21%)
Pallavi et al. in 2018 [27]	7 (40.9%)	-	12 (54.6%)	-	13 (34.5%)	-	9 (28.1%)	-	27 (27.3%)	-	44 (44.4%)	-
Gupta et al. in 2019 [16]	18 (33.96%	-	21 (39.6%)	-	9 (33.33%)	-	18 (66.66%)	-	6 (30%)	-	17 (85%)	-
Dave et al. in 2016 [19]	-	-	55 (51%)	7 (6.38%)	-	-	27 (75%)	6 (16.66%)	-	-	45 (78.94%)	18 (31.57%)

Limitations

The results from the present study need to be further strengthened and validated by multilocational and bigger sample-sized research studies to predict the severity of disease and maternal and fetal outcomes. Also, the study was conducted in a single hospital hence the results cannot be corroborated or generalized in a community setting.

## Conclusions

Serum LDH levels were significantly higher in women with preeclampsia and eclampsia compared to the normotensive pregnant women. Higher LDH levels were positively correlated with disease severity and maternal complications like placental abruption, HELLP, DIC, acute renal failure, intracranial hemorrhage, pulmonary edema, and maternal death, and for fetal complications like preterm, IUGR, APGAR at 1 minute < 7, APGAR at 5 minutes < 7, LBW, NICU admission, and IUFD.
